# From Blog to lab: testing messages to shift public opinion on gene editing

**DOI:** 10.3389/fbioe.2026.1816909

**Published:** 2026-06-17

**Authors:** Luke S. Wimpy, Brandon R. McFadden, Kathryn A. Stofer, Joy N. Rumble, Kevin M. Folta

**Affiliations:** 1 Department of Agricultural Economics and Agribusiness, University of Arkansas, Fayetteville, AR, United States; 2 Department of Agricultural Education and Communication, University of Florida, Gainesville, FL, United States; 3 Department of Agricultural Communication, Education, and Leadership, The Ohio State University, Columbus, OH, United States; 4 Horticultural Sciences Department, University of Florida, Gainesville, FL, United States

**Keywords:** biotechnology, consumer acceptance, gene editing, public perception and understanding, science communication, visual communication

## Abstract

**Introduction:**

Public acceptance of gene editing remains an important consideration for the adoption of biotechnology in agriculture and medicine. This study presents findings from the final phase of a multi-stage research project designed to identify the communication strategies most effective at shaping public opinions on the safety of gene editing.

**Methods:**

A nationally recruited sample of 3,200 U.S. respondents participated in a randomized survey experiment. Participants were assigned to view one of eight videos developed from prior focus groups and three earlier surveys conducted as part of the broader research project. The videos varied across three communication attributes: messenger (researcher or blogger), message framing (emphasizing either the number of years of biotechnology use without adverse events or the number of studies demonstrating safety), and application context (agriculture or medicine). Between-subject analyses evaluated differences in communication effectiveness across treatment conditions, with outcomes focused on opinion change and knowledge retention.

**Results:**

Respondents were generally receptive to the video interventions, suggesting that brief communication messages can influence perceptions of gene-editing safety. The application context of the video significantly affected knowledge retention, with respondents demonstrating stronger retention when the information aligned with the specific field emphasized in the communication. In contrast, differences in messenger type and message framing had comparatively limited effects on knowledge outcomes, suggesting that these communication features were less influential in shaping responses.

**Discussion:**

These findings suggest that the context in which gene editing is presented may be more important than the specific messenger or message framing for communicating biotechnology safety to the public. The results offer practical insights for science communicators, industry stakeholders, and policymakers seeking to improve public understanding and acceptance of gene editing technologies across agricultural and medical applications.

## 1 Introduction

Gene editing research has advanced significantly over the past few decades, especially with the development and application of site-directed nuclease-based approaches such as CRISPR-Cas9 (Gyngell, 2017). The agricultural and medical fields have turned to gene editing to address difficult diseases and challenges. Before gene editing, farmers and plant breeders mainly relied on traditional breeding techniques to develop new plant varieties, which could take many years (Nelson, 2020). From research laboratories to industrial seed development, gene editing technology has been shown to accelerate crop improvement and enhance its precision (Ahmad, 2023). These developments could help crop production keep up with a growing global population and improve food security. There has also been progress in gene editing within the livestock industry, as pigs have been genetically modified to become more disease-resistant and to have more desirable production traits (Whitworth et al., 2022). Continued efforts in this area could lead to healthier animals raised for meat, supporting long-term food security. Similarly, in medicine, gene editing has the potential to accelerate drug development and possibly treat genetic diseases and disabilities (e.g., Gyngell, 2017).

Extensive assessments have been conducted to evaluate the safety of gene-edited crops, and these studies show that gene-edited crops do not pose a greater health risk than conventionally bred crops through consumption (Lassoued et al., 2019). These results align with the lack of credible evidence of negative health effects from genetic engineering methods after nearly 30 years of use (NAS, 2016). However, many U.S. adults still do not consider bioengineering safe (Funk et al., 2016). The general public’s knowledge and acceptance of gene editing in both medicine and agriculture remain limited and largely negative for some (McFadden et al., 2024). Still, people wish for engagement on the issue (Scheufele et al., 2017). Since U.S. adults may buy or consume gene-edited products, increasing their awareness of and perceptions about gene editing is crucial for gaining social acceptance and enabling further progress in the field.

Consumers often associate gene editing with older genetic engineering methods, such as adding or suppressing a gene using “transgenic” technologies, commonly called “GMO” approaches. However, these technologies are different. Because traditional genetic engineering methods often carry a negative reputation, gene editing might also be viewed negatively by consumers (McFadden et al., 2021). Other research shows that many people do not understand gene editing, which can lead to hesitation in accepting gene-edited products (Stofer and Schiebel, 2017). Scientists and producers should explain the similarities and differences between gene editing, transgenic techniques, and traditional breeding techniques to aid consumer acceptance of gene editing (McFadden et al., 2021). Doing so could improve public understanding of gene editing and its risks and benefits, and give consumers the information they need to feel confident about its safety and regulation.

Consumer concerns about biotechnology have been documented in other developed areas outside the U.S. (McFadden and Smyth, 2019). In Europe, successive Eurobarometer waves have documented persistently cautious public attitudes toward agricultural biotechnology, with substantial variation in acceptance across countries (Gaskell et al., 2006; Gaskell et al., 2010). More recent work specific to gene editing and other precision-breeding techniques finds that European consumers distinguish gene editing from older transgenic methods but remain relatively skeptical (Delwaide et al., 2015; Bearth and Siegrist, 2016). Shew et al. (2018) examined consumer preferences across five countries (the U.S., Canada, Belgium, France, and Australia) and found a higher preference for gene editing than for GMO equivalents. A meta-analysis by Frewer et al. (2013) identified perceived benefit, perceived naturalness, and trust in regulators as the most consistent predictors of consumer attitudes. This body of work demonstrates that public responses vary systematically by application type. Medical and pharmaceutical applications of genetic modification have consistently attracted greater acceptance than agricultural and food applications, which are often evaluated as posing risks without commensurate personal benefits (Frewer et al, 1997; Siegrist, 2000; Gaskell et al., 2010). These application-based distinctions are not incidental; they reflect a stable public heuristic in which perceived medical necessity offsets risk aversion, whereas agricultural uses are judged against a more demanding cost-benefit calculus (Bearth and Siegrist, 2016).

Research on the most effective ways to communicate the safety of gene editing has been limited. Although open communication and transparency can lead to higher acceptance (McFadden et al., 2021), these strategies are not always sufficient. While labels have been introduced to identify products as organic, “bioengineered” (transgenic), or even gene-edited, these labels might not adequately inform people about the meaning of gene editing and its associated safety. For example, individuals might see a gene-edited label on a product and immediately return it because of pre-existing beliefs about genetically engineered products. In a sense, the labels are transparent about the product itself, but not necessarily informative about what they truly signify. Research on other scientific topics points to the role of the source (Bolsen et al., 2019; Brondi et al., 2021) or worldview (Većkalov et al., 2023). Finally, survey participants call for more research to convince them of a scientific consensus, yet we do not know how much is enough or how to frame the amount of research. Some research indicates numeric messages do increase acceptance of consensus (Myers et al., 2015).

Figuring out how to effectively communicate the meaning and safety of gene editing will be crucial in the years to come, as the technologies are already in the field and are likely to be part of many plant and human genetic improvement efforts. Therefore, we aimed to build on previous findings about U.S. consumers' concerns and combine them with their preferences for educational settings to explore which kinds of messaging influence attitude change, especially among the movable middle. Our previous research indicated unclear preferences for educational settings for messages about gene editing, so we chose video formats for testability and for relative preference and ubiquity across educational settings, including social media as a site for potential incidental exposure (Anderson et al., 2021), as well as to compare with previous qualitative results (Stofer et al., 2023). In this study, the movable middle refers to respondents who are neither strongly for nor against gene editing before watching a video intervention. This intervention could either shift respondents toward a more positive view of gene editing or have the opposite effect. This group is often of interest to researchers because it comprises a large share of the population and is more likely to be influenced (McFadden and Lusk, 2015).

The goals of this study were to examine how video intervention affects respondents’ opinions and understanding of gene editing and its applications in agriculture and medicine. Specifically, the study will analyze how different speakers, types of persuasive information, and contexts influence opinions on gene editing. Analyzing potential differences in demographic variables relative to consumer knowledge, opinion, and acceptance are also of interest for this study.

The study objectives were met by analyzing survey results on gene editing in agriculture and medicine. Respondents answered questions about gene editing in these areas to establish a baseline of public perception regarding its safety. After establishing this baseline, each respondent was randomly assigned to one of eight videos explaining the safety of gene editing to identify factors that influence or do not influence changes in their safety perceptions. Following the survey, ordered logit tests were used to compare the effects of each video, examining how speakers, content, and context influenced respondents’ beliefs and opinions about gene editing.

## 2 Materials and methods

This study was approved by the Institutional Review Board at the <<*masked for review*>> (Protocol #: 2402522897).

### 2.1 Survey overview

Respondents were asked initial questions about their knowledge and beliefs regarding gene editing and its use in agriculture and medicine. Then, each respondent was randomly shown one of eight 30-s videos focused on the safety and application of gene editing in either agriculture or medicine. After watching the video, respondents answered follow-up questions to determine whether their opinions on gene editing had changed, followed by standard demographic questions. Many questions were selected from previous studies to ensure consistency with the overall population and to identify potential shifts in public opinion over time.

The survey for this project was created using Qualtrics software and distributed to an online panel recruited and maintained by Prolific. Data were collected from 3,200 respondents in September 2024. Quota sampling was used to ensure the sample matched the U.S. population in age, ethnicity, and sex.

### 2.2 Video development

We created eight videos for the study, arranged in a 2 × 2 × 2 design. Four videos focused on the benefits of gene editing in agriculture and food applications, while the other four emphasized its benefits in medical applications. Four videos featured a speaker identifying as a blogger, and the other half featured a speaker identifying as a researcher. Additionally, four videos each contained persuasive messages claiming that either more than 1,000 studies or 30 years of research had been conducted without harm to animals or humans. We hired a professional actor from a local talent agency after screening demo reels from a pool of applicants. We selected an actor with the most neutral appearance possible, choosing a middle-aged female—neither young nor old—with a look that could not easily be associated with any specific race. The actor recorded all videos using a single script written by authors three and 5, a science communication and biotechnology expert, respectively, with only the three variables previously described differing across videos. The script for the 'Researcher, Time, Agriculture' video is as follows:

My name is [NAME], and I research food technology. Today, I want to talk about gene editing in agriculture, the technology that you may have seen as a little “bioengineered” sticker on some grocery store products. As new technologies emerge, you might wonder whether the benefits outweigh the risks. Scientific consensus shows that, in more than 30 years of independent research on food containing ingredients from bioengineered crops, there have been no verified negative effects on human or animal health. In fact, no special risks have been identified, and products containing bioengineered ingredients can be safer, more available, and more nutritious. So I’m confident in choosing foods with the bioengineered label for my family and friends, and I hope you can be too.

Refer to Table 1 for details on the wording of the other scripts. The videos were recorded in a professional studio at the University of <<*masked for review*>>, with support from experienced production staff. While our development predated their review, we followed many of the principles identified by Montes et al. (2025) when creating the scripts and producing the videos.

**TABLE 1:** Video names and scripts.

*Video name*	*Script*
Researcher_Time_Ag (RTA)	My name is [NAME], and I research food technology. Today, I want to talk about gene editing in agriculture, the technology that you may have seen as a little “bioengineered” sticker on some grocery store products. As new technologies emerge, you might wonder whether the benefits outweigh the risks. Scientific consensus shows that, in more than 30 years of independent research on food containing ingredients from bioengineered crops, there have been no verified negative effects on human or animal health. In fact, no special risks have been identified, and products containing bioengineered ingredients can be safer, more available, and more nutritious. So I’m confident in choosing foods with the bioengineered label for my family and friends, and I hope you can be too
Blogger_Time_Ag (BTA)	My name is [NAME], and I blog about food technology. Today, I want to talk about gene editing in agriculture, the technology that you may have seen as a little “bioengineered” sticker on some grocery store products. As new technologies emerge, you might wonder whether the benefits outweigh the risks. Scientific consensus shows that, in more than 30 years of independent research on food containing ingredients from bioengineered crops, there have been no verified negative effects on human or animal health. In fact, no special risks have been identified, and products containing bioengineered ingredients can be safer, more available, and more nutritious. So I’m confident in choosing foods with the bioengineered label for my family and friends, and I hope you can be too
Researcher_Studies_Ag (RSA)	My name is [NAME], and I research food technology. Today, I want to talk about gene editing in agriculture, the technology that you may have seen as a little “bioengineered” sticker on some grocery store products. As new technologies emerge, you might wonder whether the benefits outweigh the risks. Scientific consensus shows that, in more than 1,000 independent research studies on food containing ingredients from bioengineered crops, there have been no verified negative effects on human or animal health. In fact, no special risks have been identified, and products containing bioengineered ingredients can be safer, more available, and more nutritious. So I’m confident in choosing foods with the bioengineered label for my family and friends, and I hope you can be too
Blogger_Studies_Ag (BSA)	My name is [NAME], and I blog about food technology. Today, I want to talk about gene editing in agriculture, the technology that you may have seen as a little “bioengineered” sticker on some grocery store products. As new technologies emerge, you might wonder whether the benefits outweigh the risks. Scientific consensus shows that, in more than 1,000 independent research studies on food containing ingredients from bioengineered crops, there have been no verified negative effects on human or animal health. In fact, no special risks have been identified, and products containing bioengineered ingredients can be safer, more available, and more nutritious. So I’m confident in choosing foods with the bioengineered label for my family and friends, and I hope you can be too
Researcher_Time_Med (RTM)	My name is [NAME], and I research medical technology. Today, I want to talk about gene editing in medicine, the technology that you may have heard can help treat cancer and inherited genetic diseases. As new technologies emerge, you might wonder whether the benefits outweigh the risks. Scientific consensus shows that, in more than 30 years of independent research on medical applications of bioengineering, there have been no verified negative effects on human or animal health. In fact, no special risks have been identified, and medicines containing bioengineered ingredients can enhance disease treatment and prevention and be more affordable. So I’m confident in choosing bioengineered medicines and procedures for my family and friends, and I hope you can be too
Blogger_Time_Med (BTM)	My name is [NAME], and I blog about medical technology. Today, I want to talk about gene editing in medicine, the technology that you may have heard can help treat cancer and inherited genetic diseases. As new technologies emerge, you might wonder whether the benefits outweigh the risks. Scientific consensus shows that, in more than 30 years of independent research on medical applications of bioengineering, there have been no verified negative effects on human or animal health. In fact, no special risks have been identified, and medicines containing bioengineered ingredients can enhance disease treatment and prevention and be more affordable. So I’m confident in choosing bioengineered medicines and procedures for my family and friends, and I hope you can be too
Researcher_Studies_Med (RSM)	My name is [NAME], and I research medical technology. Today, I want to talk about gene editing in medicine, the technology that you may have heard can help treat cancer and inherited genetic diseases. As new technologies emerge, you might wonder whether the benefits outweigh the risks. Scientific consensus shows that, in more than 1,000 independent research studies on medical applications of bioengineering, there have been no verified negative effects on human or animal health. In fact, no special risks have been identified, and medicines containing bioengineered ingredients can enhance disease treatment and prevention and be more affordable. So I’m confident in choosing bioengineered medicines and procedures for my family and friends, and I hope you can be too
Blogger_Studies_Med (BSM)	My name is [NAME], and I blog about medical technology. Today, I want to talk about gene editing in medicine, the technology that you may have heard can help treat cancer and inherited genetic diseases. As new technologies emerge, you might wonder whether the benefits outweigh the risks. Scientific consensus shows that, in more than 1,000 independent research studies on medical applications of bioengineering, there have been no verified negative effects on human or animal health. In fact, no special risks have been identified, and medicines containing bioengineered ingredients can enhance disease treatment and prevention and be more affordable. So I’m confident in choosing bioengineered medicines and procedures for my family and friends, and I hope you can be too

We note that the scripts intentionally used the term “bioengineered” because this is the regulatory term U.S. consumers encounter on food product labels, and because the safety evidence referenced in the scripts (more than 30 years of independent research and more than 1,000 studies) is drawn primarily from the broader bioengineering/transgenic research literature rather than from gene-editing research alone. Our study, therefore, examines how opinion and knowledge shift toward the combined “gene editing/bioengineering” framing that U.S. consumers are most likely to encounter in the real world, rather than toward a narrowly defined conception of gene editing. We return to this point when discussing study limitations.

Table 2 describes the eight videos used in this study. If a characteristic is marked with an “X,” then the video contains that characteristic. The number of respondents randomized to each video is also reported.

**TABLE 2:** Video names, attributes, and number of respondents.

​	*Speaker*		*Content*		*Context*		*Number of respondents*
*Video name*	Researcher	Blogger	No negative effects in over 30 years	No negative effects in over 1,000 studies	Agriculture/Food	Medical/Medicine	
Researcher_Time_Ag (RTA)	X	​	X	​	X	​	403
Blogger_Time_Ag (BTA)	​	X	X	​	X	​	400
Researcher_Studies_Ag (RSA)	X	​	​	X	X	​	403
Blogger_Studies_Ag (BSA)	​	X	​	X	X	​	396
Researcher_Time_Med (RTM)	X	​	X	​	​	X	395
Blogger_Time_Med (BTM)	​	X	X	​	​	X	409
Researcher_Studies_Med (RSM)	X	​	​	X	​	X	397
Blogger_Studies_Med (BSM)	​	X	​	X	​	X	397

### 2.3 Survey questions

#### 2.3.1 Introductory Questions and pre-video assessments

Respondents first indicated their level of agreement or disagreement with seven statements to assess their trust in science and technology on a five-point Likert scale, ranging from “strongly disagree” to “strongly agree.” They were also asked three questions to gauge their opinions on gene editing in medicine (the same questions were asked about gene editing in agriculture). Whether the respondent received the medical or agricultural questions first was determined randomly. First, they indicated their familiarity with the technology’s use in medicine on a three-point scale: a lot, a little, or none. The next question asked about the strength of their opinion regarding the safety of gene editing in health and medical applications, with options of strong, weak, or no opinion. Responses to this question were used to create the “strength of opinion before video intervention” variable for the medical domain. The last question asked for their opinion on safety on a five-point Likert scale from “extremely safe” to “extremely unsafe.” Responses to this question were used to generate the “opinion about safety before video intervention” for medical. The same three questions were also asked about the use of gene editing in food and agriculture, and responses were used to build the “familiarity,” “strength of opinion before video intervention,” and “opinion about safety before video intervention” for agriculture.

**FIGURE A1:**
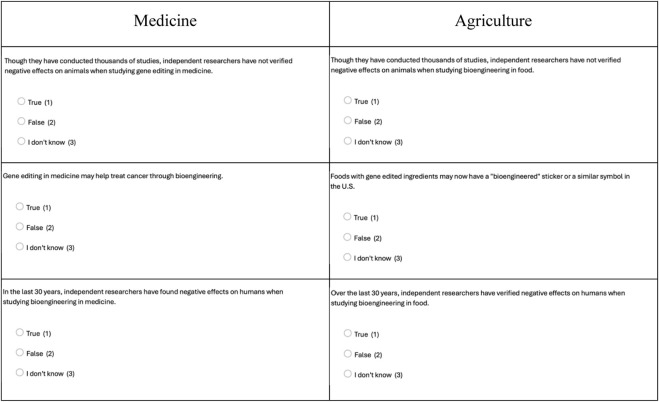
knowledge questions asked both before and after video intervention.

After the opinion questions, each respondent was then asked a series of knowledge questions to assess their actual understanding of gene editing in the real world (see Appendix Figure A1 for the specific question wording). All knowledge questions offered responses of “true,” “false,” and “I do not know.” The six knowledge questions focused on safety and specific side effects related to technology use in medical and agricultural applications, based on concerns from previous study respondents. Generally, these covered side effects that could harm animals or humans, as documented in thousands of studies over 30 years. We also asked whether food might have bioengineered labels in the U.S. and if bioengineering could be used to treat cancer. The combined responses to the questions about agriculture created the “ag knowledge before” variable, and those about medicine formed the “med knowledge before” variable.

After the initial questions, each respondent watched one randomly assigned 30-s video as described above. We uploaded the videos to a private YouTube account and embedded them into the survey. We tested the randomization to ensure approximately equal views of each video before launching the full survey. Respondents had to stay on the page for 60 s before continuing in the survey.

#### 2.3.2 Post-video assessments

Following watching the video, each respondent was asked four questions about how their opinions on gene editing may have changed in the context of both health/medical and food/agriculture. To establish the “strength of opinion after video intervention” variables for medical and agriculture, we asked whether their opinion about safety in the relevant context changed; response options were stronger, weaker, or unchanged. Next, we asked how the respondent’s opinion on the safety of gene editing changed after watching their video, with response options presented as a five-point Likert scale ranging from “extremely more safe” to “extremely less safe,” thus creating the “opinion about safety after video intervention” variables for both medical and agriculture.

We repeated the knowledge questions from before watching the video for variables “ag knowledge after” and “med knowledge after.” We then asked about their confidence in their answers to the knowledge questions on a five-point Likert scale, ranging from “very confident” to “not at all confident.” Finally, we asked how much they trusted the speaker in the video and how much they trusted the video’s content. They could respond with “not at all,” “only a little,” “somewhat,” and “quite a bit.” These two questions were asked to determine who was delivering the message in the video, and if the evidence used to support the claims in the video (like time or studies) influenced how the respondent answered.

Finally, we asked 12 items related to cultural cognition (Kahan et al., 2010) and standard demographic questions. Each respondent’s answers to the 12 cultural cognition items were used to create the “individual” and “hierarchical” variables. Several of the demographic questions were used to develop variables included in our models. Specifically, the age question was used to create the “age” variable, the gender question to make the “gender (female)” variable, the income question to establish the “income” variable, the education question to form the “education” variable, and respondents were asked to describe their area of residence, which allowed for the creation of the “density” variable. Additionally, respondents were asked which region of the U.S. they lived in, leading to the creation of the “region” variable. An ethnicity question was asked to generate the “Hispanic,” “African American,” and “White” variables.

After the basic demographic questions, respondents were asked if they had a disability. If they answered yes, they were then asked whether their disability was genetic (used to create the “genetic disability” variable). Identifying whether a disability was genetic was particularly important given the genetic focus of this study. The goal was to determine whether people with a genetic disability viewed gene editing in agriculture or medicine differently from those without one.

Lastly, respondents were asked whether they had worked in agriculture or medicine, which enabled the creation of the “ag occupation” and “med occupation” variables. Each respondent was also asked to rate how much they knew about each field compared to other people their age living in the U.S. Response options ranged in 10% increments, starting with “100% (Everyone my age knows more about the health or medical field than I do)” and ending with “0% (No one my age knows more about the health or medical field than I do).” These two questions contributed to the “subjective knowledge” variables. These four questions were included because the study’s focus is on agriculture and medicine, and to determine whether those who have worked in one of these fields or are more knowledgeable about them have different perspectives on gene editing in medicine or agriculture.

Summary statistics for respondent demographics are reported in Table 3.

**TABLE 3:** Summary statistics for demographic variables.

*Age*	*Statistic*
Mean age	49.935
*Gender (female)*	
Female	50.38%
Non-female	49.62%
*Income*	
Less than $10,000 (1)	2.88%
$10,000 to $19,999 (2)	5.38%
$20,000 to $29,999 (3)	8.34%
$30,000 to $39,999 (4)	9.03%
$40,000 to $49,999 (5)	8.25%
$50,000 to $59,999 (6)	9.84%
$60,000 to $69,999 (7)	8.31%
$70,000 to $79,999 (8)	7.88%
$80,000 to $89,999 (9)	5.34%
$90,000 to $99,999 (10)	6.03%
$100,000 to $149,999 (11)	18.91%
$150,000 or more (12)	10.09%
Mean	7.307
*Education*	
Less than high school degree (1)	0.66%
High school graduate (high school diploma or equivalent including GED) (2)	13.31%
Some college but no degree (3)	21.16%
Associate’s degree in college (2-year) (4)	11.66%
Bachelor’s degree in college (4-year) (5)	35.22%
Graduate or professional degree (MS, PhD, JD, MD) (6)	18.00%
Mean	4.21
*Trust*	
Mean trust (on a scale of 1-5)	4.31
*Density*	
Urban	27.81%
Suburban	53.06%
Rural	19.12%
*Region*	
Northeast	17.84%
Midwest	19.78%
South	44.69%
West	17.69%
*Race*	
Hispanic	5.94%
African American	15.12%
White	70.72%
*Genetic disability*	
Have genetic disability	5.75%
Do not have a genetic disability	94.25%
*Ag occupation*	
Yes	13.62%
No	86.38%
*Med occupation*	
Yes	21.69%
No	78.31%
*Ag subjective knowledge*	
100% (Everyone my age knows more about the food or agricultural field than I do) (1)	2.00%
About 90% (2)	4.72%
About 80% (3)	7.47%
About 70% (4)	10.94%
About 60% (5)	11.44%
About 50% (6)	24.88%
About 40% (7)	13.81%
About 30% (8)	13.09%
About 20% (9)	7.16%
About 10% (10)	3.94%
0% (no one my age knows more about the food or agricultural field than I do) (11)	0.56%
Mean	5.95
*Med subjective knowledge*	
100% (Everyone my age knows more about health or medical field than I do) (1)	0.69%
About 90% (2)	1.81%
About 80% (3)	3.88%
About 70% (4)	7.31%
About 60% (5)	11.31%
About 50% (6)	26.09%
About 40% (7)	15.91%
About 30% (8)	19.09%
About 20% (9)	9.66%
About 10% (10)	3.81%
0% (no one my age knows more about the health or medical field than I do) (11)	0.44%
Mean	6.52
*Individual*	
Mean individual cultural cognition score	22.84
*Hierarchical*	
Mean hierarchical cultural cognition score	16.58

n = 3,200. Coded values are represented by the values in parentheses.

### 2.4 Statistical analysis

After creating the before and after knowledge variables, the “ag knowledge change” and “med knowledge change” variables were calculated by subtracting “ag knowledge before” from “ag knowledge after,” and similarly for med variables. All statistical analyses were conducted using Stata. To verify that randomization was effective during the survey, a “tabulation” command in Stata was used for all videos, and the results confirmed proper randomization. Between-subject tests were employed to compare the effects of the videos.

Using Stata software, ordered logit testing was used to analyze the effects of each video on all knowledge variables and across all demographics. The findings of these logit tests are presented in the tables within the results section.

## 3 Results

Table 4 shows the frequency distributions for consumer familiarity with gene editing and opinions about safety before and after the video intervention. Respondents seemed to be more familiar with gene editing in agriculture than in the medical field. 91.41% of respondents indicated they had heard either a little or a lot about gene editing in agriculture, compared to only 75.38% for gene editing in the medical field. Consumer opinions are also shown in Table 4. These data indicate that more people tend to have an opinion on the safety of gene editing in agriculture than in medical use, regardless of whether their opinion is positive or negative. Regarding the strength of respondents’ opinions before the video intervention, they appear more likely to hold and strongly hold opinions about the safety of gene editing in agriculture than in the medical field.

**TABLE 4:** Summary statistics for consumer familiarity of gene editing and opinions about safety before and after video intervention.

*Familiarity*	*Agriculture*	*Medical*
Not heard (0)	8.59%	24.62%
Heard a little (1)	66.19%	63.72%
Heard a lot (2)	25.22%	11.66%
Mean	1.17	0.87
*Strength of opinion before video intervention*		
None (0)	22.50%	39.50%
Weak (1)	48.31%	42.19%
Strong (2)	29.19%	18.31%
Mean	1.07	0.79
*Strength of opinion after video intervention*		
Weaker (−1)	4.19%	3.91%
Unchanged (0)	45.84%	46.38%
Stronger (1)	49.97%	49.72%
Mean	0.46	0.46
*Opinion about safety before video intervention*		
Extremely unsafe (−2)	7.88%	4.28%
Somewhat unsafe (−1)	21.78%	16.16%
Neutral (0)	22.03%	41.78%
Somewhat safe (1)	36.16%	31.84%
Extremely safe (2)	12.16%	5.94%
Mean	0.23	0.19
*Opinion about safety after video intervention*		
Extremely less safe (−2)	5.56%	2.88%
Somewhat less safe (−1)	10.06%	4.41%
No change of opinion (0)	16.88%	34.34%
Somewhat more safe (1)	38.81%	37.78%
Extremely more safe (2)	28.69%	20.59%
Mean	0.75	0.69

n = 3,200. Responses were reverse-coded and the recoded values are represented by the values in parentheses.

After the video intervention, a change in opinion strength was also observed, with about 50% of people reporting that their opinions became stronger across both disciplines after watching the video. Favorable views on the safety of gene editing in both agriculture and medicine increased by an average of 0.52 and 0.50 points, respectively. These statistics suggest that the video intervention, regardless of which video each person watched, generally led to more positive and stronger views on the use of gene editing in agriculture and medicine.

Table 5 shows respondents’ knowledge levels about gene editing in agriculture and medicine before and after the video intervention. As seen in Table 5, the video intervention leads to improved understanding among respondents. This suggests that the videos effectively educated respondents about gene editing and its safety in agriculture and medicine. Additionally, regardless of which video each person watched or its context, knowledge seems to have increased in both areas.

**TABLE 5:** Mean number of knowledge questions answered correctly.

​	*Agriculture*	*Medicine*
Knowledge before video intervention	1.05	1.02
Knowledge after video intervention	1.49	1.44
Change in knowledge	0.43	0.42

n = 3,200. All respondents were asked the same three ag knowledge questions and three med questions before and after watching their video.

Table 6 presents the effects of each video on respondents’ knowledge of gene editing in the agricultural and medical fields, based on ordered logit tests. When examining changes in agricultural knowledge before and after the video intervention, a significant increase is observed across all agricultural-related videos, with the RSA video producing the largest increase in knowledge (1.13), indicating it was the most effective at enhancing understanding of gene editing in agriculture. For medical knowledge, a similar pattern appears, as all medical videos increased respondents' reported understanding. The RTM video resulted in the greatest gain, with an increase of 1.13. Overall, these results suggest that the video’s context—whether agricultural or medical—is a more prominent factor when it comes to influencing the knowledge that a respondent gains than the speaker (researcher or blogger) and the content (duration or number of studies).

**TABLE 6:** The effects of video information on knowledge.

Video group	Ag knowledge before	Ag knowledge after	Ag knowledge change	Med knowledge before	Med knowledge after	Med knowledge change
*Agricultural videos*						
Researcher_Time_Ag (RTA)	0.032	1.305***	1.052***	-	-	-
​	(0.130)	(0.138)	(0.133)	-	-	-
Blogger_Time_Ag (BTA)	0.101	1.152***	0.865***	−0.063	−0.208	−0.160
​	(0.130)	(0.137)	(0.132)	(0.131)	(0.131)	(0.133)
Researcher_Studies_Ag (RSA)	0.042	1.378***	1.131***	−0.088	−0.146	−0.012
​	(0.130)	(0.139)	(0.133)	(0.130)	(0.131)	(0.132)
Blogger_Studies_Ag (BSA)	−0.020	1.127***	1.005***	−0.094	−0.077	0.069
​	(0.130)	(0.136)	(0.133)	(0.130)	(0.130)	(0.133)
*Medical videos*						
Researcher_Time_Med (RTM)	-	-	-	−0.147	1.130***	1.133***
​	-	-	-	(0.130)	(0.136)	(0.134)
Blogger_Time_Med (BTM)	−0.027	−0.060	−0.023	−0.027	1.022***	0.936***
​	(0.132)	(0.133)	(0.130)	(0.130)	(0.134)	(0.132)
Researcher_Studies_Med (RSM)	−0.017	−0.034	−0.020	0.159	1.002***	0.768***
​	(0.132)	(0.133)	(0.132)	(0.131)	(0.134)	(0.134)
Blogger_Studies_Med (BSM)	−0.005	0.018	0.055	0.020	1.042***	0.950***
​	(0.131)	(0.133)	(0.131)	(0.130)	(0.136)	(0.133)
Log likelihood	−3,826	−3,417	−4,065	−3,731	−3,587	−3,957

The agricultural Researcher_Time video was used as the base in all medical column estimations, and the medical Researcher_Time video was used as the base in all agricultural column estimations. All models used 3,200 observations. ***denotes significance at the 0.01 level; values in parenthesis represent standard errors.

Table 7 shows how video intervention, knowledge level, and respondent demographics influenced opinions about the safety of gene editing in both agriculture and medicine. Each model (M1, M2, M3) presents results from different ordered logit tests across multiple variables. Model1displays the results of an ordered logit test comparing each video to respondents’ opinions on agricultural and medical gene editing after the intervention. Model 2 (M2) presents the results of an ordered logit test showing medical and agricultural opinions controlling for each video, as well as respondent knowledge variables. Model 3 (M3) displays the results of an ordered logit test on medical and agricultural opinions after the video intervention, controlling for each video, respondent knowledge, and demographics.

**TABLE 7:** The effects of video intervention, knowledge, and demographics on opinions about the safety of using gene editing.

​	*Agricultural opinions (favorability) after video*			*Medical opinions (favorability) after video*		
Independent variables	M1	M2	M3	M1	M2	M3
*Agricultural videos*						
Researcher_Time_Ag (RTA)	0.376***	0.102	0.072	-	-	-
​	(0.129)	(0.131)	(0.134)	-	-	-
Blogger_Time_Ag (BTA)	0.484***	0.250*	0.259*	0.027	0.121	0.133
​	(0.128)	(0.130)	(0.133)	(0.130)	(0.131)	(0.134)
Researcher_Studies_Ag (RSA)	0.376***	0.094	0.162	−0.163	−0.125	−0.089
​	(0.129)	(0.131)	(0.134)	(0.130)	(0.132)	(0.135)
Blogger_Studies_Ag (BSA)	0.196	−0.043	0.019	−0.168	−0.139	−0.079
​	(0.129)	(0.129)	(0.133)	(0.130)	(0.132)	(0.135)
*Medical videos*						
Researcher_Time_Med (RTM)	-	-	-	0.678***	0.397***	0.540***
​	-	-	-	(0.130)	(0.132)	(0.135)
Blogger_Time_Med (BTM)	0.243*	0.270**	0.224*	0.722***	0.483***	0.571***
​	(0.127)	(0.127)	(0.131)	(0.130)	(0.132)	(0.135)
Researcher_Studies_Med (RSM)	0.118	0.127	0.053	0.652***	0.378***	0.456***
​	(0.129)	(0.127)	(0.130)	(0.131)	(0.133)	(0.135)
Blogger_Studies_Med (BSM)	0.243*	0.262**	0.246*	0.677***	0.433***	0.548***
​	(0.129)	(0.129)	(0.131)	(0.132)	(0.134)	(0.136)
*Knowledge*						
Ag knowledge before	​	0.416***	0.340***	​	-	-
​	​	(0.050)	(0.052)	​	-	-
Ag knowledge change	​	0.540***	0.486***	​	-	-
​	​	(0.048)	(0.049)	​	-	-
Ag subjective knowledge	​	−0.016	−0.033**	​	-	-
​	​	(0.015)	(0.016)	​	-	-
Med knowledge before	​	-	-	​	0.847***	0.698***
​	​	-	-	​	(0.052)	(0.054)
Med knowledge change	​	-	-	​	0.701***	0.645***
​	​	-	-	​	(0.048)	(0.050)
Med subjective knowledge	​	-	-	​	−0.044**	−0.067***
​	​	-	-	​	(0.018)	(0.019)
*Demographics*						
Age	​	​	−0.003	​	​	−0.002
​	​	​	(0.002)	​	​	(0.002)
Gender (female)	​	​	−0.446***	​	​	−0.111
​	​	​	(0.069)	​	​	(0.070)
Income	​	​	0.011	​	​	0.016
​	​	​	(0.011)	​	​	(0.011)
Education	​	​	0.062**	​	​	0.008
​	​	​	(0.027)	​	​	(0.028)
Trust	​	​	1.188***	​	​	1.050***
​	​	​	(0.067)	​	​	(0.068)
Density	​	​	0.013	​	​	−0.034
​	​	​	(0.052)	​	​	(0.053)
Region	​	​	0.027	​	​	0.031
​	​	​	(0.034)	​	​	(0.035)
Hispanic	​	​	0.028	​	​	0.396**
​	​	​	(0.176)	​	​	(0.180)
African American	​	​	0.162	​	​	0.372**
​	​	​	(0.145)	​	​	(0.146)
White	​	​	0.106	​	​	0.125
​	​	​	(0.123)	​	​	(0.124)
Genetic disability	​	​	0.015	​	​	0.070
​	​	​	(0.096)	​	​	(0.098)
Ag occupation	​	​	−0.016	​	​	-
​	​	​	(0.098)	​	​	-
Med occupation	​	​	-	​	​	−0.016
​	​	​	-	​	​	(0.085)
Individual	​	​	−0.059***	​	​	−0.056***
​	​	​	(0.007)	​	​	(0.007)
Hierarchical	​	​	−0.008*	​	​	0.007
​	​	​	(0.005)	​	​	(0.005)
Log likelihood	−4,526	−4,460	−4,058	−4,091	−3,930	−3,671

The agricultural Researcher_Time video was used as the base in all medical model estimations, and the medical Researcher_Time video was used as the base in all agricultural model estimations. All models used 3,200 observations. ***, **, and * denote significance at the 0.01, 0.05, and 0.1 levels, respectively; values in parentheses represent standard error of the mean; M1 shows the effects that video intervention had on respondent opinion in each discipline; M2 shows the effects video intervention and knowledge levels had on respondent opinion; M3 shows the effects video intervention, knowledge levels, and demographics had on respondent opinions.

The results from Model 1 indicate that three of the four agricultural videos—excluding the BSA video—had a significant positive (more favorable) effect on respondent opinions about the use and safety of gene editing in agriculture. Meanwhile, all four medical videos significantly positively influenced opinions regarding medical applications. Regarding the impact on disciplinary opinions not addressed in the videos, both medical blogger videos had a statistically significant positive effect on agricultural opinions, but only at the 0.1 significance level. In contrast, videos focused on agriculture did not influence opinions in the medical context.

Based on Model 2 results, the BTA, BTM, and BSM showed a statistically significant positive change in respondents' opinions of gene-editing safety in the agricultural field compared with the RTM baseline video. It also appears that the level of knowledge a respondent had before watching their video significantly influenced their opinions on gene editing in the respective field after viewing. This trend suggests that individuals with more prior knowledge of gene editing within a discipline tend to have a more favorable view of its use in that field. Additionally, all three videos that impacted agricultural opinions featured a blogger as the speaker, and two of them had a medical context. A possible reason is that respondents initially held a more positive outlook on gene editing in agriculture, making it less likely that an agricultural video would significantly strengthen their opinion compared to the medical videos.

Medical opinion after video intervention in Model 2 shows that all medical-related videos led to a statistically significant positive increase in medical opinions about gene editing compared to the base video of RTA. This indicates that all medical videos foster a more favorable view of gene editing in the medical field. The statistical significance observed for “Med knowledge before” and “Med knowledge change” indicates that the video intervention increased medical knowledge about gene editing. “Med subjective knowledge” is also statistically significant in Model 2, but because the coefficient is negative, it suggests that video intervention was associated with a less favorable outlook on the use of gene editing in the medical field among individuals with higher levels of subjective medical knowledge.

Similar to Model 2, Model 3 shows that agricultural opinions on gene editing seem to increase—meaning they become more favorable—after watching the BTA, BTM, and BSM videos. The significance of “Ag knowledge before” and “Ag knowledge change” indicates that the video intervention prompted a more positive view of gene editing in agriculture. The negative coefficient for “Ag subjective knowledge” suggests that those who believed they knew more than others about agriculture tended to have a less favorable opinion of gene editing after viewing a video. The statistical significance (p < 0.01) of the “Gender” variable and the coefficient of −0.446 show that women are less likely to hold a more favorable opinion, even after the video intervention. “Education” and “Trust” have positive, statistically significant coefficients, indicating that individuals with higher levels of education are more likely to view gene editing in agriculture favorably after watching the videos. The same trend applies to trust in science. The cultural cognition test shows that individuals who are more “Individual” versus community-oriented or “Hierarchical” versus egalitarian tend to have a less favorable opinion of gene editing in agriculture after seeing the videos.

Regarding medical opinions based on Model 3, all medical videos showed a positive, statistically significant change in respondents’ opinions of gene editing in the medical field compared to the baseline video. “Med knowledge before” and “Med knowledge change” both show statistically significant coefficients, indicating that the video intervention led to a more favorable view of gene editing in medicine. Similar to the agricultural findings from Model 3, “Med subjective knowledge” appears to show that those who believed they knew more about medicine had a less favorable opinion after the video intervention. The coefficients for demographics “Trust,” “Hispanic,” and “African American” are all positive and statistically significant in this model, while gender was not, suggesting that those with higher trust in science, or who are Hispanic or African American, tended to have a more positive opinion on gene editing in medicine after watching the video. Lastly, individuals with a more “Individualistic” perspective of the world (per the cultural cognition test) tend to have a more negative outlook on gene editing in the medical field after viewing the video.

## 4 Discussion

This study on how video elements, knowledge, and demographic characteristics influence opinions on gene editing showed that U.S. adults generally viewed gene editing in both agricultural and medical fields as neutral to favorable and perceived the technology as neutral to safe, even before watching any videos. Before the video intervention, more respondents had a favorable view of gene editing in agriculture than of its medical uses.

After videos were introduced, respondents’ opinions and knowledge of gene editing increased in both disciplines. The videos appeared to have not only raised the amount of knowledge respondents gained but also either strengthened their opinions on the safety of gene editing across both disciplines or resulted in minimal change. On average, both video contexts produced a net positive shift in perceived safety. However, approximately 16% of respondents reported that gene editing in agriculture was less safe or extremely less safe after the agricultural videos, compared with approximately 7% after the medical videos (see Table 4). This pattern seems to indicate that opinions about the agricultural applications were more polarized than those about its medical applications, with the agricultural videos reinforcing strong positions in both directions.

All agricultural videos led to increased knowledge of agricultural gene editing, just as all medical videos enhanced understanding of gene editing in the medical field. It is worth noting that no clear pattern emerged regarding which speaker or content was more effective at boosting knowledge, as shown in Table 6. These results support the idea that, if the video’s context matches the post-video questions, higher knowledge retention in that field is likely after watching one of the videos, whether in agriculture or medicine. In simple terms, whether the speaker was a blogger or a researcher, and whether the content involved studies or a period without negative incidents, seemed less important than the overall message that there is no evidence of negative side effects.

Results on opinion change follow the same trend as those of knowledge change in medical applications, but the same cannot be said for agricultural opinions. Only the BTA video showed a statistically significant change in respondents’ opinions on gene editing in agriculture across all three models, though the researcher videos also increased opinions positively in Model 1. A possible explanation is that respondents were more accepting and familiar with gene editing in agriculture even before the video intervention, so the opportunity to increase respondents’ opinion of gene editing in agriculture was smaller. Additionally, when other variables were added in Models 2 and 3, these new variables may have captured some of the change in opinion that explains the lack of significance for video effects within agriculture for researcher videos in those models. In models 2 and 3, the negative coefficients and their statistical significance for “Ag Subjective Knowledge” and “Med Subjective Knowledge” can be interpreted as those who claim to have more subjective knowledge within a discipline were more likely to have less favorable opinions within the same discipline after video intervention. In a sense, this suggests that those who feel they know a lot about gene editing within a given field may not actually know as much as they think—a trend that has been observed in other studies of a similar nature as well (Fernbach, et al., 2019). Model 3 shows that females (p < 0.01) are more likely to have less favorable opinions of gene editing use in agriculture. Additionally, those with a higher level of education tend to have a more favorable outlook on gene editing in agriculture. Looking at the results for Hispanics and African American respondents in Model 3 regarding the medical discipline, it appears these groups are more accepting of gene editing in medicine. Future research could explore the reasoning behind these demographic patterns.

Our findings on opinions of safety among U.S. adults contrast prior science communication work on related terminology and technology, especially *genetically modified organism (GMO)* (Ruth and Rumble, 2019; Scott et al., 2018; Shew et al., 2018), but are in line with our prior surveys on *gene editing* as both a more precise and perhaps less inflammatory technology with potential benefits to society. We also infer, based on the pre-intervention knowledge surveys, a general lack of understanding of the technology, consistent with previous surveys on familiarity with the technology (Calabrese et al., 2021; Stofer and Schiebel, 2017). Given the proportions of favorable opinions toward gene editing technology relative to related genetic engineering technologies, we suggest science communicators focus on increasing engagement with gene editing to build even more confidence among consumers, especially through videos and incidental social media exposure (Anderson et al., 2021). Our videos specifically suggest the potential to stress the benefits and the extensive evidence supporting safety. Finally, respondents reporting a disability represent approximately 5.75% of the sample, with the genetic-disability subgroup smaller still; this subsample is descriptive only and does not support multi-way factorial analysis. Nevertheless, within these limits the reported presence of a disability did not predict opinion change in the videos. This may mean that this community warrants particular attention, especially in the medical context. It could be that this population is unaware of the benefits gene editing could offer for genetic disabilities, due to confusion about terminology, or that these messages do not address specific concerns about the technology among disabled people (e.g., Feliú-Mójer, 2020; Hoffman-Andrews et al., 2019).

Open questions remain about the difference in opinions between medical and agricultural applications of gene editing. Future science communication and public opinion research could focus on why agricultural gene editing is more accepted and better known than medical gene editing, particularly by examining social values related to perceived benefits (Davies et al., 2009). We could also explore why videos in a single disciplinary context did not change opinions or knowledge in the related context about the same technology, even though our videos presented evidence in just one discipline each time. Such findings point to the need to discuss gene editing and other scientific and technological advances across a wide range of contexts and with diverse members of society to best promote adoption.

While we provided science communicators with preliminary evidence that both the number of studies and the years of research influence opinions, there is a great deal more to be done to understand the limits of such evidence, particularly what is a minimum to influence opinions more in line with scientific understanding of safety. On the other hand, sharing this work with scientists can help them understand community concerns that must be addressed to maintain trust in science and promote the uptake of scientifically-backed societal- and individual-level solutions. Future research and engagement can help understand the needs of communities with particular interests, such as those with disabilities, especially those with genetically related disabilities; farmers and merchants who might sell products with genetically edited ingredients; and livestock managers who could use gene editing to combat disease in their animals. Centering those communities and partnering with them for co-creation of science communication and engagement messaging responsive to ethical and other concerns, as well as research, is a vital next step to maximize uptake of the technology and its benefits while attending to ethical issues (Boardman, 2019; Fatima et al., 2025; Feliú-Mójer, 2020; Stamell, 2017).

While these videos proved effective for increasing respondents’ awareness of the safety of gene editing, they did not provide much detail about what gene editing actually looks like in agriculture and medicine. The term “gene editing” is quite broad and can seem intimidating or hard to understand, especially to those unfamiliar with scientific research. To address this, future science communication research could explore how people set boundaries for gene editing and what practices, products, and applications they are more willing to accept or reject in both agriculture and medicine. This could be achieved through methods such as surveys, small focus groups, or face-to-face interviews in controlled settings, where different uses and products derived from gene editing are presented to respondents to gauge which aspects are viewed more favorably and which still carry negative perceptions. In particular, we recommend more qualitative work, as suggested by analogous work in nanotechnology (Davies et al., 2009), especially to guard against framing knowledge and opinion as a lack among U.S. adults or as a fixed set of attitudes over time (Davies et al., 2009).

Science communicators and engagement professionals can use our results to build their own messaging about gene-editing technology to encourage conversation about the risks, benefits, and other concerns that may keep people opposed to a technology that could benefit society in at least some cases. In the meantime, science communicators can be aware of the potential for confusion around the different technologies of modification and their applications, and do their best to frame their work deliberately so as to be on the same page with their communities and audiences. Videos and other materials that draw comparisons between medical editing and agricultural editing contexts may help audiences with a specific contextual background build upon more favorable or more malleable opinions. We must also ensure that science communication and engagement move beyond dialogue to actually influence policy (Davies et al., 2009), rather than simply building support for existing policy, especially to address concerns of people who may be consequence-insensitive and uninterested in the technology’s benefits for other reasons. Finally, as science communication continues to explore multimodality, especially for contested issues (Metag and Wintterlin, 2026), our work contributes to understanding video production and persuasive scientific messaging development using experimental methods.

Overall, our results contribute to the science-communication literature by showing that short (30-s) videos that combine a benefits frame with an absence-of-risks frame produced measurable positive shifts in both knowledge and opinions about gene editing among U.S. adults. Across all eight videos, respondents’ knowledge of gene editing increased in the matching context (agricultural or medical), and their mean perceived safety shifted positively by approximately 0.53 points (agriculture) and 0.50 points (medicine) on a five-point scale. Within the video design, context (agriculture vs. medicine) had a larger and more consistent effect on knowledge retention and opinion change than either messenger (researcher vs. blogger) or message content (years vs. number of studies).

Several limitations should be noted when interpreting these findings. First, as discussed in Section 2.2, the video scripts used the term “bioengineered” and drew on a broader bioengineering evidence base. Therefore, our findings describe opinion and knowledge shifts toward the combined gene-editing/bioengineering framing rather than toward the more narrowly defined conception of gene editing. Experimentally separating these framings is an important direction for future work. Second, the study used a between-subjects design in which each respondent viewed only one of eight videos, and we did not include a no-video control condition. Between-video comparisons, therefore, isolate the relative effects of messenger, message, and context but cannot determine a pure “video vs. no video” effect. Third, the disability subsample is too small to support factorial analysis of messenger, message, and context within that subgroup, and the disability-related results are descriptive only. Finally, opinion and knowledge were assessed immediately after a single 30-s video; longer-term retention and real-world behavioral change remain open questions for future research.

## Data Availability

The datasets presented in this study can be found in online repositories. The names of the repository/repositories and accession number(s) can be found below: https://osf.io/4qp2g/overview?view_only=738936e545424598a5dd4d7c076441ee.
